# Post-treatment Lyme disease symptoms score: Developing a new tool for research

**DOI:** 10.1371/journal.pone.0225012

**Published:** 2019-11-11

**Authors:** Siu P. Turk, Keith Lumbard, Kelly Liepshutz, Carla Williams, Linden Hu, Kenneth Dardick, Gary P. Wormser, Joshua Norville, Carol Scavarda, Donna McKenna, Dean Follmann, Adriana Marques

**Affiliations:** 1 Laboratory of Clinical Immunology and Microbiology, National Institute of Allergy and Infectious Diseases, National Institutes of Health, Bethesda, Maryland, United States of America; 2 Clinical Monitoring Research Program Directorate, Frederick National Laboratory for Cancer Research, Frederick, Maryland, United States of America; 3 Leidos Biomedical Research, Inc., Clinical Services Program, Frederick National Laboratory for Cancer Research, Frederick, Maryland, United States of America; 4 Tufts University School of Medicine, Boston, Massachusetts, United States of America; 5 Mansfield Family Practice, Storrs, Connecticut, United States of America; 6 Infectious Diseases, New York Medical College, Valhalla, New York, United States of America; 7 Biostatistics Research Branch, National Institute of Allergy and Infectious Diseases, National Institutes of Health, Bethesda, Maryland, United States of America; University of Kentucky College of Medicine, UNITED STATES

## Abstract

Some patients have residual non-specific symptoms after therapy for Lyme disease, referred to as post-treatment Lyme disease symptoms or syndrome, depending on whether there is functional impairment. A standardized test battery was used to characterize a diverse group of Lyme disease patients with and without residual symptoms. There was a strong correlation between sleep disturbance and certain other symptoms such as fatigue, pain, anxiety, and cognitive complaints. Results were subjected to a Logistic Regression model using the Neuro-QoL Fatigue t-score together with Short Form-36 Physical Functioning scale and Mental Health component scores; and to a Decision Tree model using only the QoL Fatigue t-score. The Logistic Regression model had an accuracy of 97% and Decision Tree model had an accuracy of 93%, when compared with clinical categorization. The Logistic Regression and Decision Tree models were then applied to a separate cohort. Both models performed with high sensitivity (90%), but moderate specificity (62%). The overall accuracy was 74%. Agreement between 2 time points, separated by a mean of 4 months, was 89% using the Decision Tree model and 87% with the Logistic Regression model. These models are simple and can help to quantitate the level of symptom severity in post-treatment Lyme disease symptoms. More research is needed to increase the specificity of the models, exploring additional approaches that could potentially strengthen an operational definition for post-treatment Lyme disease symptoms. Evaluation of how sleep disturbance, fatigue, pain and cognitive complains interrelate can potentially lead to new interventions that will improve the overall health of these patients.

## Introduction

Lyme disease is the most common tick-borne illness in the United States and Europe. Lyme disease usually begins with the characteristic skin lesion, erythema migrans. From the site of inoculation, *Borrelia burgdorferi* may spread and cause neurologic, cardiac and/or rheumatologic manifestations.

While the objective signs of disease typically resolve following antibiotic therapy, subjective symptoms may persist for some patients. The frequency of residual subjective non-specific symptoms 6 to 24 months after therapy has ranged between 0 to 23% for patients with erythema migrans [1–13]. These persistent or relapsing symptoms are referred to as post-treatment Lyme disease (PTLD) symptoms, and if they cause a substantial reduction in previous levels of activity, PTLD syndrome [4]. The pathogenesis of these symptoms is unknown.

Research involving patients with PTLD symptoms has included descriptions of subjective symptoms as an entry criteria [14–20]. Only a few studies have focused on a standardized approach to capture symptoms and functional impact [21–23]. In this study, we aimed to characterize patients with and without PTLD symptoms to develop statistical models to be used in research studies. Results were used to develop a Logistic Regression model and a Decision Tree model. These models were then applied to a separate cohort. Both models were highly consistent with the clinical categorization and showed excellent agreement between 2 separate time points. These models are simple and can help to quantitate the level of symptom severity in post-treatment Lyme disease symptoms.

## Methods

### Study protocol

Written informed consent was obtained from all participants. All participants in the Development Cohort and 5 participants in the Validation cohort were enrolled under protocol NCT02446626, a total of 17 PTLDs and 17 recovered subjects. The study was approved by the institutional review board of the National Institute of Allergy and Infectious Diseases, National Institutes of Health (Bethesda, MD), Tufts Medical Center (Boston, MA) and New York Medical College (Valhalla, NY). The National Institute of Allergy and Infectious Diseases’ institutional review board serves as the review board for the Mansfield Family Practice under a reliance agreement. Other participants in the Validation Cohort were enrolled under protocol NCT00028080 and protocol NCT00001539. Both studies were approved by the institutional review board of the National Institute of Allergy and Infectious Diseases, National Institutes of Health (Bethesda, MD).

All participants were adults, acquired Lyme disease in the US and fulfilled the case definition of confirmed or probable Lyme disease [24]. Participants in the recovered group completed antibiotic therapy at least 12 months before evaluation, to allow for waning of symptoms [3, 6]. Participants with PTLD symptoms received at least one course of antibiotic therapy and had persistent or relapsing symptoms that began within 6 months of treatment and continued at least intermittently for at least 12 months post initial treatment. Data included in the study were collected from May 2016 to July 2018.

### Questionnaires

Self-administered questionnaires included the Short Form-36 (SF-36) version 2, Fatigue Severity Scale, the Patient Self-report Survey for the Assessment of Fibromyalgia, Neuro-QoL Cognition Function SF v2.0 (Neuro-QoL Cognition), Neuro-QoL Fatigue SF v1.0 (Neuro-QoL Fatigue), Neuro-QoL Sleep Disturbance SF v1.0 (Neuro-QoL Sleep), Neuro-QoL Anxiety SF v1.0 (Neuro-QoL Anxiety), Neuro-QoL Ability to Participate in Social Roles and Activities SF v1.0 (Neuro-QoL Social Participation), Neuro-QoL Emotional and Behavioral Dyscontrol SF v1.0 (Neuro-QoL Emotional), Neuro-QoL Positive Affect and Well-Being SF v1.0 (Neuro-QoL Positive Affect), and Neuro-QoL Satisfaction with Social Roles and Activities SF v1.1 (Neuro-QoL Social Satisfaction).

SF-36 scores were tabulated using QualityMetric, Inc. software. SF-36 applies norm-based scoring where the US general population has a mean score of 50 with a standard deviation (SD) of 10 [25]. The scores are grouped into 8 subscales: physical functioning, role limitations due to physical health, bodily pain, general health, vitality, social functioning, role limitations due to emotional health, and mental health. These scores are aggregated to a Physical Health component score and a Mental Health component score [26–29].

Neuro-QoL uses T-score metric where a clinical or general population has a mean score of 50 with SD of 10 [30, 31]. The measures Neuro-QoL Emotional, Neuro-QoL Sleep, and Neuro-QoL Fatigue were calibrated using a clinical population of patients with epilepsy, stroke, amyotrophic lateral sclerosis, multiple sclerosis, or Parkinson’s disease. All other Neuro-QoL measures used in this study utilized the general population for calibration [31, 32]. The mean score of the Fatigue Severity Scale [33] was used to compare the groups. The Assessment of Fibromyalgia score was calculated by adding the total of the 19-item Widespread Pain Index score and the two Symptoms Severity questions scores [34]. Clinicians reviewed a standardized symptom assessment and categorized the participants as recovered or PTLD symptoms. For PTLDs, it was required that the physician judge the symptoms to be possibly, probably or definitely related to Lyme disease, and to be significant enough to cause a decline of the patient’s quality of life compared with before the patient became ill from Lyme disease. No pre-set criteria (for example, a questionnaire score) was required for the assessment.

### Statistical methods

Independent sample t-tests were used to compare questionnaire variables between groups. All tests were two-sided and conducted at the α = 0.05 level. Pearson correlation coefficients were calculated to assess the association between variables. Fisher’s exact test was used to compare proportions between groups. Prediction models included logistic regression and a Decision Tree approach. Fitting methods included the Lasso, Firth’s method and backward selection. The decision tree model was fit under a conditional inference paradigm via the party package in R with default settings. All analysis was performed with the R programming language. The lower and upper limits of the 95% confidence interval for a proportion were calculated according to Newcombe, using the Wilson procedure with a correction for continuity [35]. Comparison of fatigue severity scale, physical health component and mental health component mean scores between studies were performed using two-sample t-tests with a Bonferroni correction based on the number of studies being compared to our study. For comparison of the SF-36 subscales between our study and results from Aucott et at. [22], a two-sample t-test was used.

## Results

### Development cohort participants characteristics

The cohort used to develop the model (the Development cohort) included 14 recovered and 15 individuals with PTLD symptoms (Table 1). The mean age was 59 years, with a preponderance of males in both groups. The PTLD symptoms group reported a median of 4 complaints, the most common being fatigue (13/15) and concentration/memory changes (11/15). Participants in the recovered group reported a median of 1 complaint. The recovered group more often had erythema migrans (8/14) as the main presentation of the infection, compared with PTLD symptoms (5/15). Participants in the recovered group had received a median of 1 course of antibiotics, with 2 of the 14 individuals being treated with intravenous antibiotic therapy. The PTLD symptoms group had received a median of 3 antibiotic courses, with 8 of 15 receiving intravenous antibiotic therapy. The antibiotic courses varied in duration, with most individuals receiving between 2 and 4 weeks, however 4 PTLD symptoms received courses lasting over 6 weeks.

**Table 1:** Study participant characteristics.

Characteristics	Development Cohort		Validation Cohort	
	PTLDs	Recovered	PTLDs	Recovered
**N**	15	14	10	13
**Age (mean years)**	59	59	61	52
**Female gender–no. (%)**	6 (40)	4 (29)	3 (27)	6 (46)
Lyme Disease Presentation*—no. (%)				
**Single Erythema Migrans**	4 (27)	8 (57)	1 (9)	6 (46)
**Multiple Erythema Migrans**	1 (7)	0	0	3 (23)
**Flu-like illness with seroconversion**	2 (13)	1 (7)	3 (27)	1 (8)
**Acute Neuroborreliosis**	4 (27)	2 (14)	0	3 (23)
**Carditis**	0	1 (7)	0	0
**Arthritis**	2 (13)	2 (14)	4 (36)	0
**Late Neuroborreliosis**	2 (13)	0	2 (20)	0
Single or Multiple Erythema Migrans**	9	9	4	10
Previous Antibiotic Treatment***				
**Number of oral courses—median (range)**	2 (0–6)	1 (1)	2 (1–2)	1 (1–3)
**Number of IV courses—median (range)**	1 (0–4)	0 (0–1)	1 (0–1)	0 (0–1)
**Time from Suspected Infection to Therapy**				
**median days (range)**	24 (3–840)	16 (1–300)	82 (1–1,825)	10 (1–45)
**Current Symptoms—no. (%)**				
**Fatigue**	13 (87)	1 (7)	8 (80)	2 (15)
**Concentration/memory changes**	11 (73)	1 (7)	7 (70)	2 (15)
**Arthralgia**	5 (33)	5 (35)	8 (80)	4 (30.7)
**Myalgia**	3 (20)	0	4 (40)	1 (7.6)
**Headache**	4 (27)	1 (7)	4 (40)	5 (38)
**Paresthesias**	4 (27)	0	5 (50)	2 (15)
**Word finding difficulties**	6 (40)	1 (7)	2 (20)	1 (7.6)
**Alterations in behavior/mood**	5 (33)	0	3 (30)	2 (15)
**Sleep disturbance**	5 (33)	1 (7)	1 (10)	3 (23)
**Stiff neck**	3 (20)	1 (7)	2 (20)	1 (7.6)
**Dizziness/lightheadedness**	1 (7)	0	3 (30)	1 (7.6)
**Tinnitus**	1 (7)	3 (21)	0	2 (15)
**Night sweats**	0	0	1 (10)	0

*Main manifestation of Lyme disease.

**Total number of patients who had erythema migrans or multiple erythema migrans as a manifestation of Lyme disease.

***The duration of antibiotic treatment for each course varied between 2 to 4 weeks.

PTLDs: post-treatment Lyme disease symptoms.

### Validation cohort participant characteristics

The validation cohort included 23 participants (10 PTLD symptoms and 13 recovered). The mean age of the PTLD symptoms group was 61 years, compared with 52 years in the recovered group (t-test *P* = 0.06). Males comprised 70% of the PTLD symptoms group and 54% of the recovered group. The recovered group again had a higher number of patients with erythema migrans as the main manifestation (9/13 compared with 1/10 in the PTLD symptoms group). The PTLD symptoms group reported a median of 5 symptoms versus a median of 2 symptoms in the recovered group. The most common symptoms in the PTLD symptoms group were fatigue (8/10), concentration/memory changes (7/10) and arthralgias (8/10). Participants in the recovered group received a median of 1 course of antibiotics, with 2 individuals receiving a single course of intravenous ceftriaxone therapy. PTLD symptoms patients received a median of 2 courses of antibiotics, with 7 individuals receiving a single course of intravenous ceftriaxone therapy. Most individuals received antibiotic courses lasting between 2 and 4 weeks, but 5 PTLD symptoms received courses lasting more than 6 weeks.

While the two cohorts had no noteworthy demographic differences, the clinical manifestations were more diverse in the PTLD symptoms group. Overall, for both cohorts, the frequency of non-erythema migrans manifestations of Lyme disease was higher in the subjects with PTLD symptoms (19/25) compared with recovered subjects (10/27), a difference that was significant (Fisher’s exact test *P* = 0.006). However, presence of erythema migrans as part of the illness was not significantly different between the two groups (13/25 vs 19/27, Fisher’s exact test *P* = 0.2547). The difference between the PTLD symptoms and recovered groups regarding time from suspected infection to treatment was not statistically significant (two-sample T-test p-value = 0.07). There was also no correlation between questionnaire scores and time from suspected infection to treatment (S1 Table).

### Characterization and correlation of symptoms

**Fig 1:**
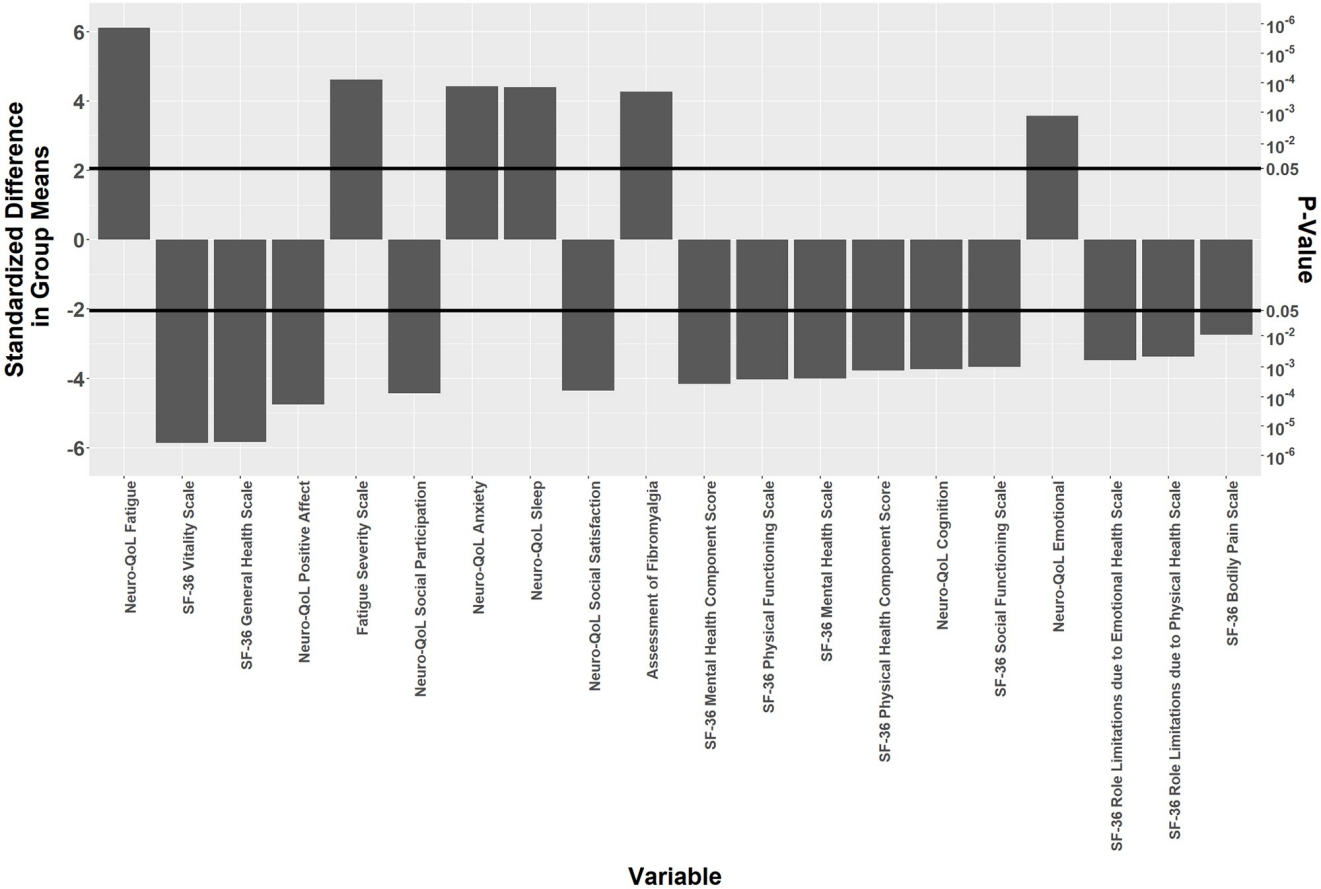
Symptom scales comparison and correlation. The difference between the two groups for a given variable was analyzed by a two-sided t-test. The standardized difference in group means is the difference between the mean of the Post-Treatment Lyme disease (PTLD) symptoms group minus the mean of the recovered group, divided by its standard error. This standardization is done so that variables measured on different scales can be compared, and the strength of evidence for a difference between groups can be quantified. Values above 0 on the y-axis indicate higher mean scores for the PTLD symptoms group, while values below 0 indicate higher mean scores for the recovered group. P-values are given to indicate the statistical magnitude of the effect.

**Fig 2:**
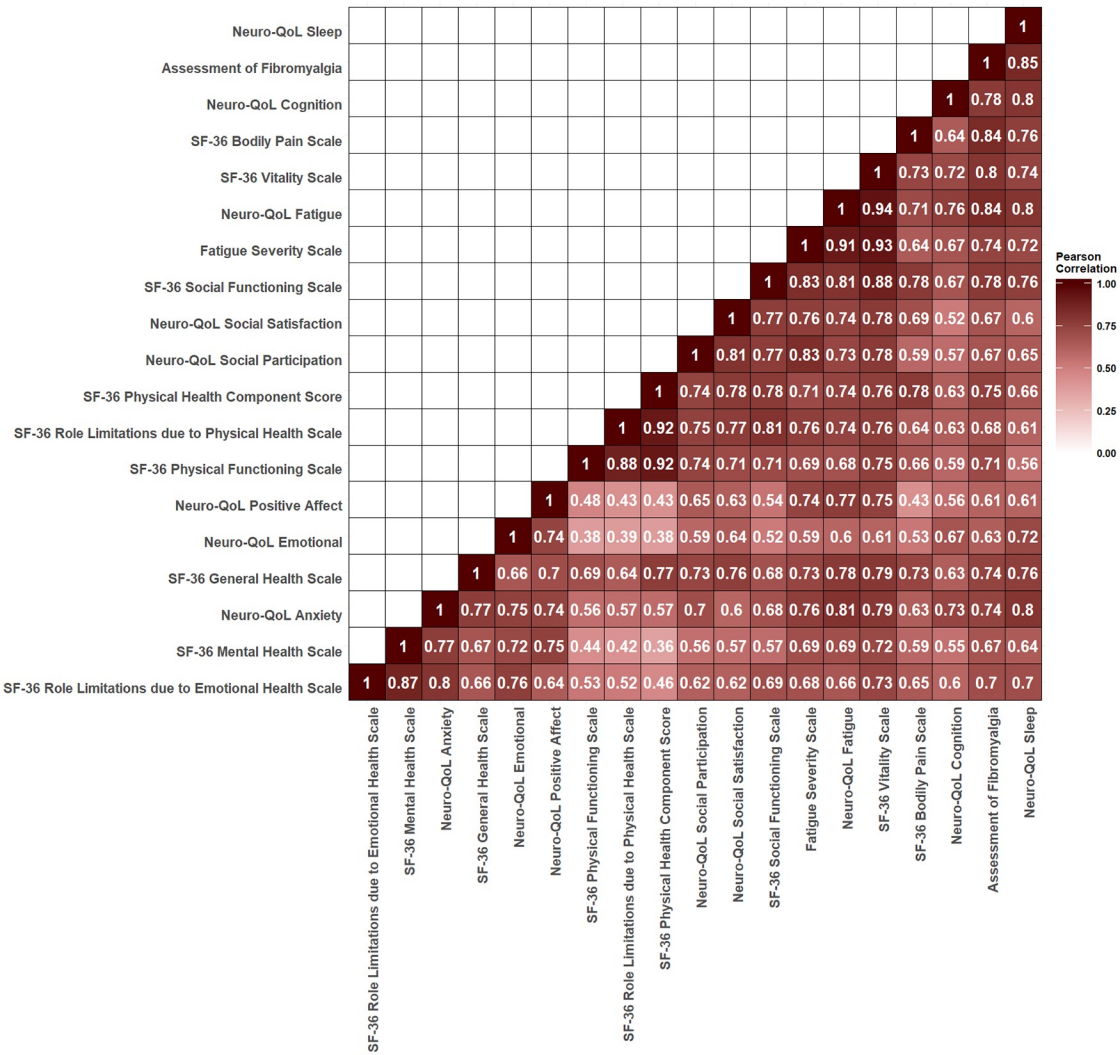
Correlation Analysis Between Symptom Scales. Scores were analyzed for pairwise covariation by the Pearson correlation analysis. All correlation coefficients with *P* < 0.05 except for the correlation between the Short Form-36 version 2 (SF-36) Physical Health Component score and the SF-36 Mental Health subscale, which had a *P* = 0.053.

We first examined if the surveys could discriminate between PTLD symptoms and recovered patients in the development cohort. In order that variables measured on different scales could be compared, the difference between the two groups for a given variable, divided by its standard error, were analyzed by two-sided t-test. We found significant differences between the 2 groups for all variables. The most significantly different variable to the least significant are shown in Fig 1. When the scores were analyzed for associations, there were strong correlations among most of the 20 variables (Fig 2). As expected, scores measuring the same domain were the most highly correlated. Clustered together were Neuro-QoL Fatigue, Fatigue Severity Scale, and SF-36 Vitality subscale, all measures of fatigue, with a correlation coefficient above 0.85. Similarly, SF-36 Bodily Pain subscale and Assessment of Fibromyalgia score, measures of bodily pain, had a correlation coefficient of 0.84. The SF-36 Mental Health and SF-36 Role Limitations due to Emotional Health subscales, measures of mental health, and the SF-36 Mental Health Component score were also highly correlated (correlation coefficient above 0.85). There were also strong correlations within variables from different domains (r≥ 0.8). For example, sleep disturbance correlated with the Assessment of Fibromyalgia score, as well as with Neuro-QoL Anxiety. The Neuro-QoL Fatigue score was also highly correlated with measures of fatigue (Neuro-QoL Fatigue and SF-36 Vitality subscale).

### Model development

Next, we wanted to develop a model that could be used in PTLD symptoms research. Because logistic regression is affected by collinearity in small samples, we tried various modeling techniques to alleviate some of those issues. These included Lasso, stepwise variable selection, Firth’s method, a combination of stepwise selection and Firth’s method, and a Decision Tree. From these techniques, the Firth version of the model, initially selected by using standard backwards selection, was chosen as our tentative logistic model.

The Logistic Regression model uses the Neuro-QoL Fatigue t-score (QoLFatigue), the SF-36 Physical Functioning norm-based score (PF) and the SF-36 Mental Health component score (MCS) to calculate a logistic predicted probability of PTLD symptoms using the equation

logit(Pr(Y=PTLDs))=49.66+0.14×(QoLFatigue)-0.24×(PF)-0.76×(MCS)

**Fig 3:**
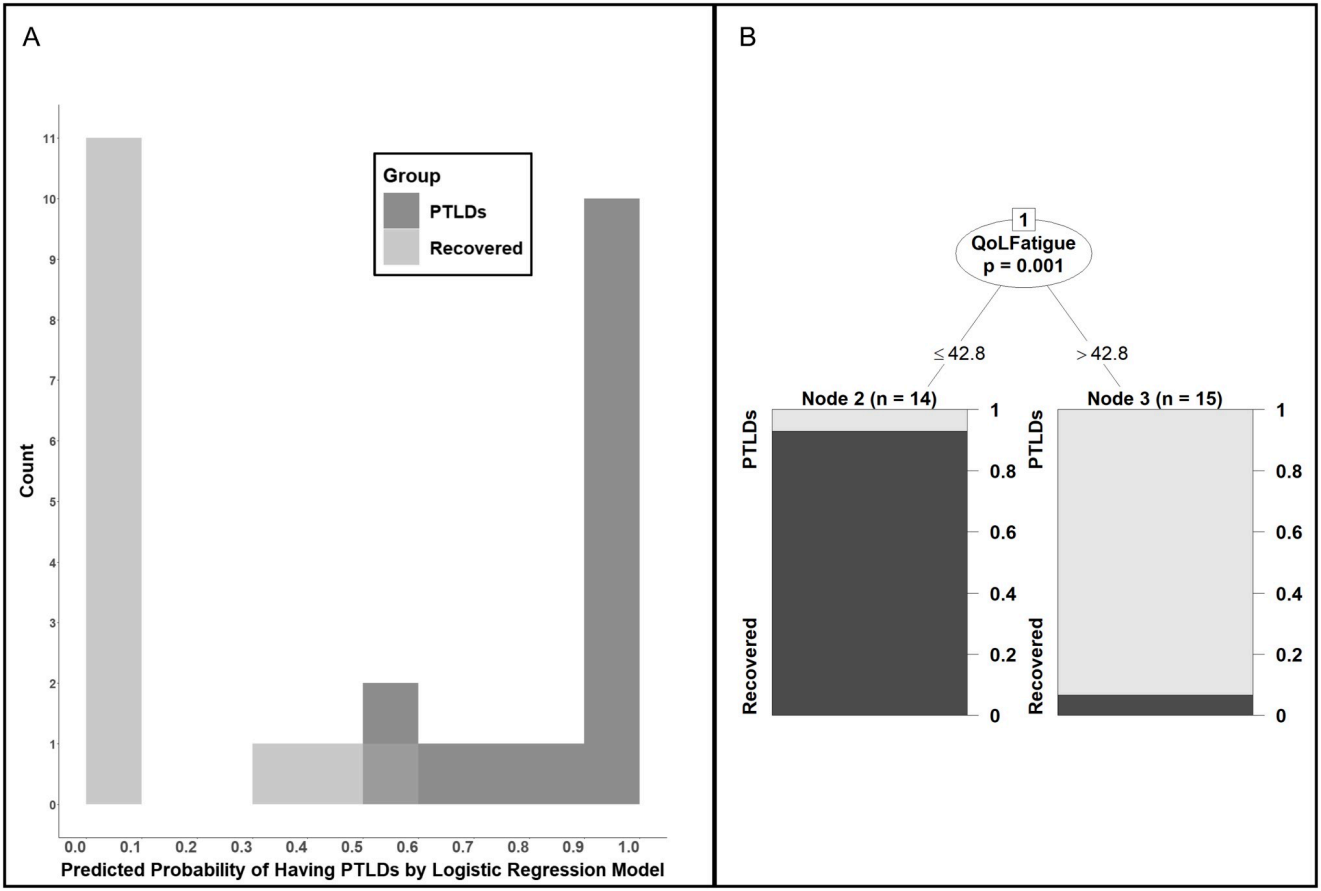
Post-treatment Lyme disease symptoms models. (A)The Logistic Regression model uses the Neuro-QoL Fatigue t-score (*Qol Fatigue*), the Physical Functioning score (*PF*) and the SF-36 Mental Health component score (*MCS*) to calculate a logistic predicted probability of post-treatment Lyme disease symptoms (*PTLDs*). The equation is: logit(Pr(Y=PTLDs))=49.66+0.14×(QoLFatigue)-0.24×(PF)-0.76×(MCS). (B) The Decision Tree model categorized QoL Fatigue t-scores ≤ 42.8 into the recovered group and QoL Fatigue t-scores > 42.8 into the post-treatment Lyme disease symptoms (PTLDs) group.

Participants with probability < 0.5 were categorized in the recovered group and ≥ 0.5 were categorized in the PTLD symptoms group (Fig 3A). To approach the question from a different perspective, we also fit a Decision Tree model. This model separated the groups using only the Neuro-QoL Fatigue t-score. Using the recovered group as the control, the Decision Tree model categorized Neuro-QoL Fatigue t-scores ≤ 42.8 into the recovered group and Neuro-QoL Fatigue t-scores > 42.8 into the PTLDs group (Fig 3B). The Logistic Regression model was 97% consistent with the clinical assessment categorization (accuracy), had a sensitivity of 100% and specificity of 93%. The Decision Tree model’s sensitivity, specificity and accuracy were all 93% (Table 2).

**Table 2:** Logistic Regression and Decision Tree Models performance by cohort.

Model	Dataset	Sensitivity (95% CI)	Specificity (95% CI)	Accuracy (95% CI)
**Logistic Regression**	Development Cohort	100% (74.65 to 100)	93% (64.2 to 99.6)	97% (82.2 to 99.9)
	Validation Cohort	90% (54.1 to 99.5)	62% (32.3 to 84.9)	74% (51.3 to 88.9)
	All	96% (77.7 to 99.8)	78% (57.3 to 90.6)	87% (73.6 to 93.9)
**Decision Tree**	Development Cohort	93% (66 to 99.6)	93% (64.2 to 99.6)	93% (75.8 to 98.8)
	Validation Cohort	90% (54.1 to 99.5)	62% (32.3 to 84.9)	74% (51.3 to 87.4)
	All	92% (72.5 to 98.6)	78% (57.3 to 90.6)	85% (71.4 to 92.7)

The lower and upper limits of the 95% confidence interval (CI) for a proportion were calculated according to Newcombe, using the Wilson procedure with a correction for continuity [35].

### Model validation

The Logistic Regression and Decision Tree model models were then applied to a new cohort of 10 PTLD symptoms and 13 recovered participants (the validation cohort). Both models performed with high sensitivity (90%), but moderate specificity (62%) in this new cohort (Table 2). Overall accuracy was 74% compared to the clinical assessment categorization. A clinical review of recovered individuals misclassified as PTLD symptoms in the models indicated that comorbidities unrelated to Lyme disease were affecting the scores. Factors cited included stress related to work and personal relationships, and underlying mood disorder that preceded Lyme disease. One PTLD symptoms individual was misclassified as Recovered using the Decision Tree model, with a Neuro-QoL Fatigue t-score of 34.1. One PTLD symptoms individual was misclassified as Recovered with the Logistic Regression model, with a predictive probability score of 0.42.

### Model performance

For additional evaluation, the Logistic Regression and the Decision Tree model models were estimated using all 52 participants and the predictions compared with the clinical categorization. The merged data were 87% consistent with the clinical categorization using the Logistic Regression model, and 85% using the Decision Tree model (Table 2). Table 3 shows a comparison of the mean scores used in this analysis. The mean Neuro-QoL Fatigue t-scores of 51.13 for our PTLD symptoms participants is equivalent to the mean level of fatigue using a clinical population with neurological disorders [31, 32]. The mean Neuro-QoL Fatigue t-scores in our recovered participants was 38.11.

**Table 3:** Comparison of the mean scores used in the mean scores used in the Logistic Regression and Decision Tree Models between the validation and development cohorts.

	PTLD symptoms			Recovered		
	Development	Validation	p-value	Development	Validation	p-value
**SF-36 MHC score (SD)**	47.71 (-10.11)	42.19 (-13.24)	0.28	58.84 (-2.16)	53.08 (-6.42)	0.008
**SF-36 PF subscale score (SD)**	47.59 (-8)	45.87 (-11.32)	0.68	56.31 (-2.45)	56.07 (-1.94)	0.78
**Neuro-QoL Fatigue t-score (SD)**	51.1 (-6.53)	51.18 (-8.48)	0.98	37.19 (-5.72)	39.09 (-7.28)	0.46

A higher score in the Neuro-QoL Fatigue represents more symptoms and a worse score of the concept being measured (e.g., more fatigue). For both the Physical Functioning (PF) subscale and Mental Health Component (MHC) scores, a lower score represents worse symptoms. Fisher’s exact test was used to compare proportions between groups. PTLDs: post-treatment Lyme disease. SF-36: Short Form-36 version 2.

### Model performance and stability over time

To assess the stability of the participants’ phenotypes, we compared a 2^nd^ data point to the initially analyzed data point. Of the 52 participants from both cohorts, 45 had a 2^nd^ data point available for this analysis (21 PTLD symptoms and 24 recovered). The range between the 2 data points was 1–7 months, with a mean and median intervals of 4 months and 3 months, respectively. The agreement between the two data points was 89% (40/45) using the Decision Tree model and 87% (39/45) using the Logistic Regression model. When we reviewed the information from the participants with discordant results, waxing and waning severity of symptoms was the explanation in 4 PTLD symptoms participants, while conditions unrelated to Lyme disease contributed to worsening scores for 3 recovered participants. The resolution of fatigue unrelated to Lyme disease between the two timepoints was the explanation in another recovered participant.

### Comparison with other studies

**Fig 4:**
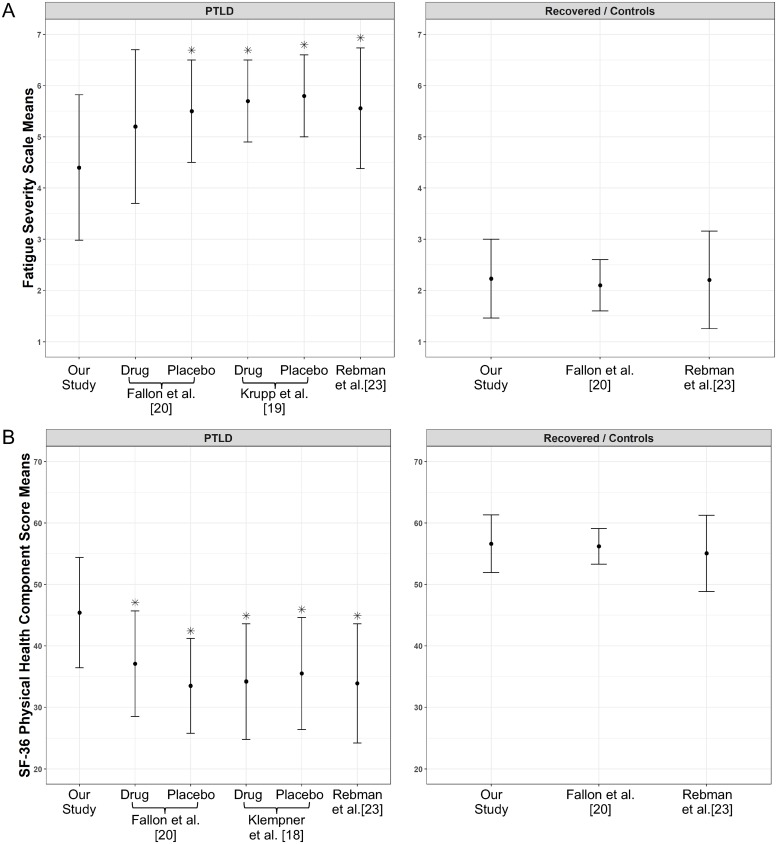
Comparison with other post-treatment Lyme disease symptoms or syndrome studies. We compared scores from our study with the scores from 4 studies (Klempner et al.[18], Krupp et al.[19], Fallon et al.[20] Rebman et al.[23]). The Fatigue Severity Scale and modified Fatigue Severity Scale mean scores, and the Physical Health component score of the Short form 36 and SF-36 v2 were compared, and the baseline assessment point was chosen for interventional studies. *Denotes statistical significance when compared to our study with a two-sample T-test at the alpha = 0.05 level after applying a Bonferroni correction based on the number of studies being compared to our study. PTLD: Post-Treatment Lyme Disease Symptoms or Syndrome.

Because the majority of participants (in both groups) in this study were participating in protocol NCT02446626, there was a concern that the PTLD symptoms group could represent a more severe end of the clinical spectrum. To address this question, we compared scores from our study with scores from 3 interventional retreatment trials [8–20] and a PTLD syndrome study [23]. The Fatigue Severity Scale and modified Fatigue Severity Scale mean scores [36], and the Physical Health component and a Mental Health component scores of the SF-36 and SF-36 version 2 were compared, and the baseline assessment point was chosen for interventional studies [18–20]. The results are shown in Fig 4 and S1 Fig. When compared with patients enrolled in the Krupp et al. study [19], that had an entry criteria of severe fatigue, our PTLD symptoms patients had a significantly lower fatigue score (i.e., less fatigue). They also had lower fatigue scores when compared with Rebman et al.[23] and with the placebo group (but not the antibiotic group) in the Fallon et al. study [20] (Fig 4A). For the Physical Health component score, our PTLD symptoms group had significantly better scores than patients evaluated under Klempner et al. [18], Fallon et al.[20], and Rebman et al.[23] (Fig 4B). There were no significant differences for the Mental Health component scores between our cohort and patients in these 3 studies [18, 20, 23] (S1 Fig). Our recovered group had scores for all 3 measures that were similar to healthy controls in both the Fallon et al. [20] and Rebman et al.[23] studies (Fig 4 and S1 Fig). Therefore, our PTLD symptoms cohort appears to have a less severe phenotype that many of the patients enrolled in prior PTLD syndrome studies.

Our PTLD symptoms subjects appear to more closely resemble those described in Aucott et al. study [22]. This study, which prospectively evaluated symptomatic patients with erythema migrans, reported the mean values for the SF-36v2 subscales for patients with and without PTLD syndrome (PTLD syndrome was defined as the presence of self-reported new-onset fatigue, widespread musculoskeletal pain, or neurocognitive difficulties) at 6 months post therapy. There were no significant differences between PTLD syndrome patients in that study and the PTLD symptoms patients in our study for all but one subscale (Table 4). The General Health subscale mean value was significantly lower in our PTLD symptoms patients, indicating greater impairment. In comparison, our recovered group had significantly better scores in 5 of 8 subscales when compared with patients without PTLD syndrome from that study (Table 5).

**Table 4:** Comparison of the Short Form-36 Version 2 Subscales for patients with post-treatment Lyme disease symptoms.

	This study	Aucott et al. [22]	
Short Form-36 version 2 Subscales	PTLD symptoms mean (SD)	PTLDS positive mean (SD)	Two-sample T-test *P* value
**Bodily Pain**	45.66 (9.51)	48.11 (12.45)	0.465
**General Health**	44.65 (10.13)	51.79 (9.42)	0.017
**Mental Health**	46.47 (11.64)	49.59 (6.97)	0.269
**Physical Functioning**	46.9 (9.28)	49.03 (9.35)	0.444
**Role Limitations due to Emotional Health**	45.72 (10.64)	48.49 (7.87)	0.317
**Role Limitations due to Physical Health**	44.4 (10.33)	40.81 (10.67)	0.255
**Social Functioning**	45.91 (10.08)	42.67 (12.18)	0.337
**Vitality**	42.62 (10.98)	45.22 (13.23)	0.478

PTLDS: Post-treatment Lyme disease syndrome.

**Table 5:** Comparison of the Short Form-36 Version 2 Subscales for patients without post-treatment Lyme disease symptoms.

	This study	Aucott et al. [22]	
Short Form-36 version 2 Subscales	Recovered mean (SD)	PTLDS negative mean (SD)	Two-sample T-test *P* value
**Bodily Pain**	54.76 (5.57)	52.87 (9.84)	0.313
**General Health**	59.98 (6.23)	55.22 (5.84)	0.003
**Mental Health**	55.81 (6.3)	54.85 (5.40)	0.517
**Physical Functioning**	56.19 (2.18)	53.70 (5.14)	0.007
**Role Limitations due to Emotional Health**	55.01 (2.9)	52.59 (8.58)	0.098
**Role Limitations due to Physical Health**	55.83 (3.65)	49.13 (8.87)	<0.001
**Social Functioning**	56.6 (1.81)	50.28 (9.12)	<0.001
**Vitality**	58.76 (6.74)	53.13 (10.94)	0.01

PTLD: Post-treatment Lyme disease.

## Discussion

Developing an operational model for patients with symptoms after treatment of Lyme disease will help to better describe this clinically defined population. This is relevant when comparing outcomes and other data from different studies and may advance research on this condition. One question is how to quantify the severity of symptoms that would fulfill the criteria of “severe enough to cause a substantial reduction in previous levels of activity” of the PTLD syndrome research case definition proposed by the Infectious Diseases Society of America [4]. The determination of whether an individual has a substantial reduction in previous levels of activity is left to the self-assessment of the patient, and/or to an evaluation by the study investigator, which could in theory diverge substantially across different patients and different practitioners, a topic that should be systematically investigated. An approach using validated and easily accessible survey tools would be highly desirable to systematically quantitate the severity level of symptoms in individuals identified as having PTLD symptoms. One limitation, however, of this approach, as illustrated in this study, is in the evaluation of study subjects who have developed co-morbidities completely unrelated to Lyme disease.

In our study, we have expanded on efforts made by other investigators to better characterize and define patients regarded as having PTLD symptoms. First, we used multiple patient reported measures to evaluate the most common complaints in PTLD symptoms patients and have compared these findings with those of individuals who recovered from Lyme disease. We have evaluated the same study subjects at different time points to assess the level of consistency. A Logistic Regression model using the scores of the Neuro-QoL Fatigue together with the SF-36 Physical Functioning subscale and Mental Health component score was chosen to best discriminate between the two groups, showing a consistency of 97% with the assigned group.

One of our aims was to investigate tools that would be freely available to the public. The SF-36, developed as part of the Medical Outcomes Study [26], is a quality of life instrument [37] that has been used in many studies of Lyme disease. Studies from Europe have used SF-36 or RAND-36 [38–42], while US studies used SF-36 [5, 18–20, 28, 43–46], but more recently the SF-36v2 [21–23, 47, 48]. The original version is freely available as RAND-36 [49], but the SF-36 (which differs from RAND-36 in the method of scoring) and second version of the SF-36 (SF-36v2) [25] are copyrighted. Because of the costs and difficulties of using the SF-36v2, it would be preferable to avoid it if possible. For that reason, our study wanted also to investigate models without this instrument. We therefore chose a Decision Tree model which separated the groups using only the Neuro-QoL Fatigue t-score. This model was 93% consistent with the clinical group.

These models were then applied to a new cohort, and while both performed with high sensitivity (90%), there was lower specificity (62%), and accuracy was reduced to 74%. When both datasets were analyzed together, the overall accuracy is 87% for the Logistic Regression model and 85% for the Decision Tree model. Therefore, these models may be helpful for research in Lyme disease, but there is room for improvement, particularly regarding the specificity of both models (78%). This moderate specificity reflects the challenge of measuring complex multifaceted subjective symptoms, that can be caused by and/or attributed to different conditions, and occur frequently in the general population [50]. The models use measures reported directly by the participant, with no specific attribution of the symptoms, and no interpretation of their responses by the study team. Attribution of the symptoms to a specific condition could be a possible way to increase specificity, but there is a conundrum in attributing or accepting an attribution (or not) of non-specific symptoms to a condition, either by the patient or by the practitioner. The attribution itself adds a degree of non-specificity, as several factors may play a role in this choice. Attribution by practitioners will rely on their clinical judgment and may vary among different assessors. The same is true for attribution by the participant, as there can be marked differences in the way individuals interpret and assign symptoms [51]. All of these concerns should reinforce the need for research to identify quantifiable biomarkers unique to PTLD symptoms.

An important limitation of our study is that the majority of subjects were from a referred sample population. Although the control participants were similarly based on a referred population, recovery is a binary event, whereas the presence of residual symptoms can vary in type and in severity. A comparison of our results with previous studies showed greater similarity to that of an outcome study of prospectively enrolled patients with erythema migrans who had residual symptoms (who were evaluated at just 6 months after the diagnosis of Lyme disease) [22] than to patients with PTLD symptoms enrolled into retreatment trials [18–20] or into a PTLD syndrome referral study [23]. In the latter studies the patients were more severely impaired than those in our study.

Another study limitation is that there was a disproportionately higher frequency of non-erythema migrans manifestations of Lyme disease in the PTLD syndrome subject groups compared with the recovered subjects. The high degree of diversity of the PTLD symptoms subjects overall may be a strength of this study. Future studies of assessment tools should purposefully incorporate a wide diversity of patients with PTLD symptoms. In addition, further studies should evaluate consecutively identified Lyme disease patients and include substantial numbers of patients with both erythema migrans and non-erythema migrans clinical manifestations, to reduce the potential impact of referral bias [21].

Of note, our study showed a strong correlation between sleep disturbance, fatigue, pain and anxiety, as well as sleep disturbance with cognitive complains. Sleep quality is known to be negatively impacted by pain, and sleep and pain can influence each other in a bidirectional way [52, 53]. Sleep disturbances can contribute to pain chronification and disability [54]. Fatigue severity is also associated with poor sleep quality. Poor sleep and sleep deprivation have negative effects on attentional capacity, working memory, emotional processing, and learning [55]. These findings are corroborated by another study showing that PTLD symptoms patients have significantly greater fatigue, pain and sleep disturbance than controls [23]. The importance of sleep is also shown in a study of patients with early Lyme disease followed for 1-year post treatment. Sleep quality remained affected in the small group of patients with PTLD symptoms for up to 1 year [56].

In conclusion, our models are simple and may help to quantitate symptom severity in patients with PTLD symptoms. More research is needed to increase the specificity of the models, exploring additional measures that will strengthen an operational definition for PTLD symptoms. Our results corroborate the need to characterize and identify the etiology of poor sleep in patients with PTLD symptoms, and to optimize interventions to improve sleep quality. Evaluation of how sleep disturbance, fatigue, pain and cognitive complains interrelate can potentially lead to new interventions that will improve the overall health of these patients.

## Supporting information

S1 Fig Comparison with other post-treatment Lyme disease symptoms or syndrome studies. We compared the Mental Health component scores from our study with the scores from 3 studies (Klempner et al.[18], Fallon et al.[20] Rebman et al.[23]) using two-sample T-test at the alpha = 0.05 level after applying a Bonferroni correction. The baseline assessment point was chosen for interventional studies. There were no significant differences for the Mental Health component scores between our cohort and patients in these 3 studies. Our recovered group had scores similar to healthy controls in both the Fallon et al. [20] and Rebman et al.[23] studies. PTLD: Post-Treatment Lyme Disease Symptoms or Syndrome.
(TIF)

S1 Table Correlation Analysis Between Symptom Scales scores and time from suspected infection to treatment. (DOCX)

S2 Table Questionnaires scores. (XLSX)
