# Supplementary figures and images for: Post-treatment Lyme disease symptoms score: Developing a new tool for research
Source: PLoS One. 2019 Nov 11;14(11):e0225012. doi: 10.1371/journal.pone.0225012 (PMC6844481; doi:10.1371/journal.pone.0225012)

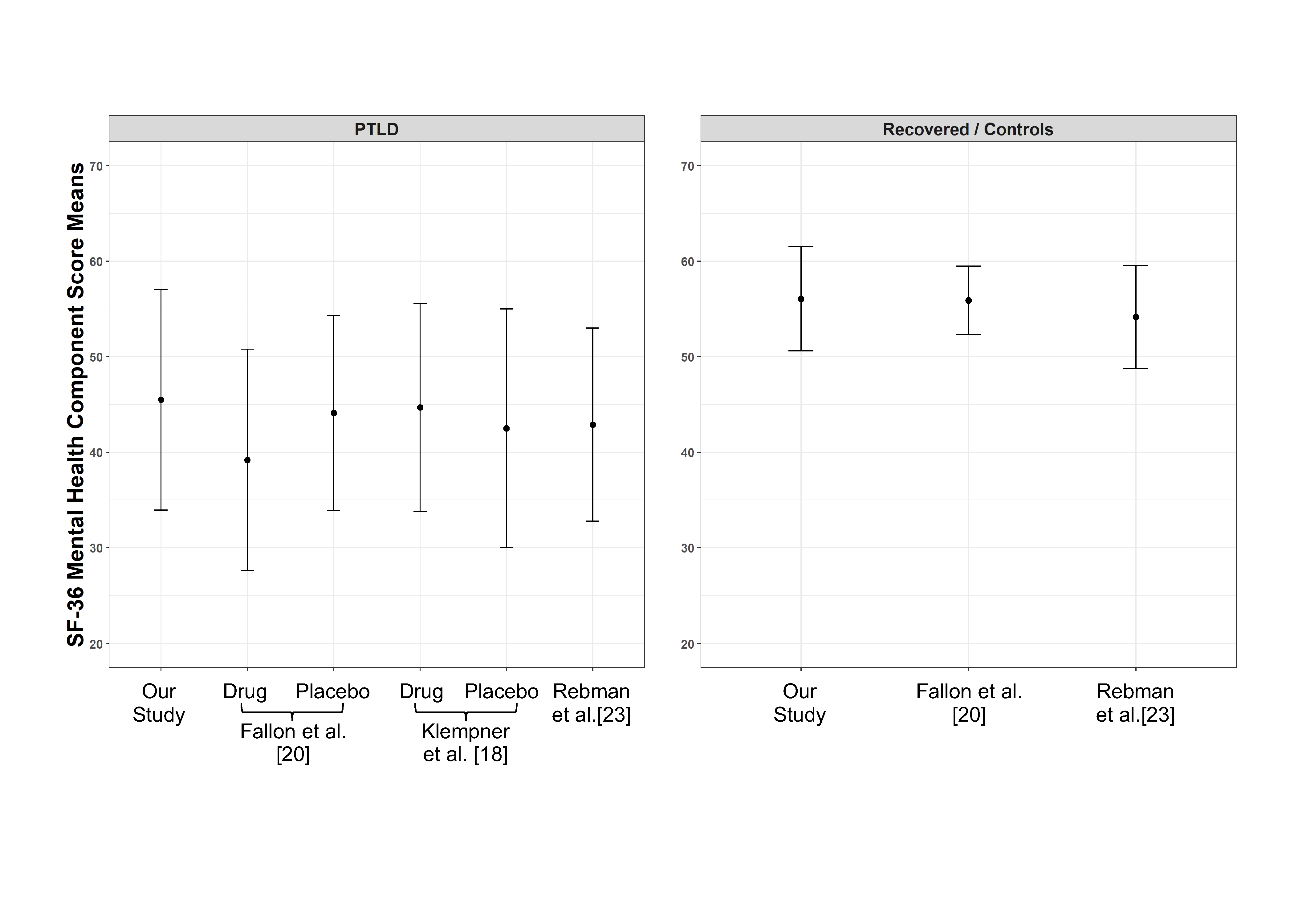

Supplement: S1 Fig — We compared the Mental Health component scores from our study with the scores from 3 studies (Klempner et al.[18], Fallon et al.[20] Rebman et al.[23]) using two-sample T-test at the alpha = 0.05 level after applying a Bonferroni correction. The baseline assessment point was chosen for interventional studies. There were no significant differences for the Mental Health component scores between our cohort and patients in these 3 studies. Our recovered group had scores similar to healthy controls in both the Fallon et al. [20] and Rebman et al.[23] studies. PTLD: Post-Treatment Lyme Disease Symptoms or Syndrome. (TIF) [file pone.0225012.s001.tif]
