# Supplementary material for: Post-treatment Lyme disease symptoms score: Developing a new tool for research
Source: PLoS One. 2019 Nov 11;14(11):e0225012. doi: 10.1371/journal.pone.0225012 (PMC6844481; doi:10.1371/journal.pone.0225012)
Supplement: S1 Table — (DOCX) [file pone.0225012.s002.docx]

**S1 Table. Correlation Analysis Between Symptom Scales Scores and Time from Suspected Infection to Treatment.**

| **Variable** | **Pearson Correlation** | **P-Value** |
| --- | --- | --- |
| Assessment of Fibromyalgia | -0.053 | 0.792 |
| Fatigue Severity Scale | 0.088 | 0.65 |
| Neuro-QoL Anxiety | 0.187 | 0.331 |
| Neuro-QoL Cognition | -0.06 | 0.756 |
| Neuro-QoL Emotional | 0.031 | 0.873 |
| Neuro-QoL Fatigue | 0.042 | 0.831 |
| Neuro-QoL Positive Affect | -0.09 | 0.643 |
| Neuro-QoL Sleep | 0.018 | 0.928 |
| Neuro-QoL Social Participation | -0.148 | 0.444 |
| Neuro-QoL Social Satisfaction | 0.052 | 0.789 |
| SF-36 Physical Health Component Score | -0.056 | 0.772 |
| SF-36 Mental Health Component Score | -0.081 | 0.677 |
| SF-36 Bodily Pain Scale | 0.152 | 0.432 |
| SF-36 General Health Scale | -0.189 | 0.327 |
| SF-36 Mental Health Scale | -0.024 | 0.903 |
| SF-36 Physical Functioning Scale | -0.166 | 0.389 |
| SF-36 Role Limitations due to Emotional Health Scale | -0.04 | 0.837 |
| SF-36 Role Limitations due to Physical Health Scale | 0.016 | 0.936 |
| SF-36 Social Functioning Scale | -0.093 | 0.631 |
| SF-36 Vitality Scale | -0.164 | 0.397 |

There were no significant correlations between the symptom scale scores and the time periods from suspected infection to treatment by Pearson correlation analysis.
